# Internalized stigma in working adults with bipolar disorder : an analysis of contributing risk factors

**DOI:** 10.1192/j.eurpsy.2026.10699

**Published:** 2026-07-31

**Authors:** F. E. Boujmil, G. Bahri, Y. Yaich, G. Amri, Y. Tebourbi, S. Chemingui, M. Mersni, D. Brahim, M. Bani, R. Ghachem, N. Ladhari

**Affiliations:** 1 Occupational health department, Charles Nicole Hospital; 2 Psychiatry B department, Razi Hospital, Tunis, Tunisia

## Abstract

**Introduction:**

Bipolar disorder (BD) is a chronic psychiatric condition characterized by recurrent mood relapses. It represents the 6^th^ leading cause of disability according to the World Health Organization. During the clinical course, patients with BD may experience internalized stigma (IS). Within a working population, it may contribute in frequent absences and professional exclusion. Its prevalence remains under-assessed despite the availability of validated instruments for its measurement.

**Objectives:**

To assess the overall IS among working adults with BD using a validated questionnaire and to identify its main associated risk factors.

**Methods:**

This cross-sectional study was conducted over a one-year period (2024-2025) among consenting BD patients who were referred for a fitness-for-work evaluation at Charles Nicole Hospital in Tunisia. Socio-demographic, medical and professional data were collected using a self-administered questionnaire. Self-stigma was assessed using the Internalized Stigma of Mental Illness (ISMI) scale, a validated instrument comprising 29 items and five dimensions rated on a four-point Likert scale. A total score ≥ 2.5 was fixed to indicate clinically significant IS.

**Results:**

A total of 106 patients were enrolled. The mean age was 43.8 ± 10.3 years with a gender ratio of 0.53 (F/H). Fifty six percent (n=59) were married and had at least one child. The majority had an average socio-economic status (72.6%). Among the patients, 41.5% (n=44) were active smokers and 12.3% (n=13) misused alcohol. The most common occupational categories were nurses (18.9%) and laborers (14.2%). The average length of service was 17.41 ± 8.7 years. Only 9.4% of patients (n=10) had a history of redundancy. Amid the patients, 33.9% (n=36) had atypical working hours. A history of long-term sick leave for psychiatric reasons was present in 88.7% of cases (n=94). Sixty-eight patients were being treated for type 2 BD (64.2%) with an average age of onset of 29.7 ± 4.9 years. The mean number of mood episodes was 6.5 ± 3.5. The average length of hospitalization was 6.4 ± 14 days. The majority of patients (n=93, 86.9%) were receiving anticonvulsants and atypical antipsychotics. The mean total ISMI was 2,58 ± 0,87. More than half of our patients (56,6%) were self-stigmatized. Social withdrawal and alienation were found in 78,3% and 55,7% of cases, followed by assimilation of stereotypes (43,4%), discrimination (39,6%) and resistance to stigma (32,1%). Univariate analysis has revealed statistically significant associations between higher self-stigma scores and gender (p = 0,038), single or divorced status (p = 0,012), low socio-economic level (p = 0,002) and atypical working hours (p <0,001).

**Conclusions:**

Preventive strategies including workplace accommodations and psychoeducation are essential to reduce stigma and support functional outcomes.

**Disclosure of Interest:**

None Declared

